# Bis(triphenyl­phosphine-κ*P*)(tropolonato-κ^2^ *O*,*O*′)silver(I) dichloro­methane solvate

**DOI:** 10.1107/S1600536809002785

**Published:** 2009-01-28

**Authors:** Gideon Steyl, Tania N. Hill

**Affiliations:** a Department of Chemistry, University of the Free State, Bloemfontein 9300, South Africa

## Abstract

The title compound, [Ag(C_7_H_5_O_2_)(C_18_H_15_P)_2_]·CH_2_Cl_2_, crystallizes with a distorted tetra­hedral geometry about the Ag^I^ atom, defined by two O atoms from one tropolonate ligand and two P atoms from two triphenyl­phosphine ligands. It is an example of a new type of tropolone derivative that has not been characterized *via* solid-state methods.

## Related literature

For general background, see: Crous *et al.* (2005 ▶); Dewar (1945 ▶). For structurally related oxalate derivatives, see: Dean *et al.* (2001 ▶). For related diketonate complexes, see: Hill & Steyl (2008 ▶); Steyl (2006 ▶).
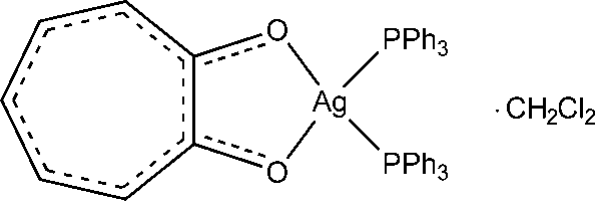


## Experimental

### 

#### Crystal data

[Ag(C_7_H_5_O_2_)(C_18_H_15_P)_2_]·CH_2_Cl_2_
*M* *_r_* = 838.45
Triclinic, 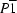

*a* = 12.0175 (3) Å
*b* = 12.9925 (4) Å
*c* = 13.8394 (7) Å
α = 100.487 (2)°
β = 93.760 (2)°
γ = 116.809 (1)°
*V* = 1869.33 (12) Å^3^
*Z* = 2
Mo *K*α radiation
μ = 0.81 mm^−1^
*T* = 100 (2) K
0.19 × 0.11 × 0.08 mm

#### Data collection

Bruker APEXII CCD diffractometer
Absorption correction: multi-scan (*SADABS*; Bruker, 2001 ▶) *T*_min_ = 0.862, *T*_max_ = 0.938
33544 measured reflections
8178 independent reflections
6931 reflections with *I* > 2σ(*I*)
*R*_int_ = 0.046

#### Refinement

*R*[*F* ^2^ > 2σ(*F* ^2^)] = 0.030
*wR*(*F* ^2^) = 0.070
*S* = 1.05
8178 reflections
460 parameters
H-atom parameters constrained
Δρ_max_ = 0.51 e Å^−3^
Δρ_min_ = −0.41 e Å^−3^

### 

Data collection: *APEX2* (Bruker, 2007 ▶); cell refinement: *SAINT-Plus* (Bruker, 2007 ▶); data reduction: *SAINT-Plus*; program(s) used to solve structure: *SHELXS97* (Sheldrick, 2008 ▶); program(s) used to refine structure: *SHELXL97* (Sheldrick, 2008 ▶); molecular graphics: *DIAMOND* (Brandenburg & Putz, 1999 ▶); software used to prepare material for publication: *SHELXL97*.

## Supplementary Material

Crystal structure: contains datablocks I, global. DOI: 10.1107/S1600536809002785/hy2179sup1.cif

Structure factors: contains datablocks I. DOI: 10.1107/S1600536809002785/hy2179Isup2.hkl

Additional supplementary materials:  crystallographic information; 3D view; checkCIF report

## Figures and Tables

**Table:** 

Ag—O1	2.3612 (16)
Ag—O2	2.3342 (16)
Ag—P1	2.4070 (6)
Ag—P2	2.4981 (5)

**Table:** 

O2—Ag—O1	68.67 (6)
O2—Ag—P1	121.43 (4)
O1—Ag—P1	128.35 (4)
O2—Ag—P2	103.97 (4)
O1—Ag—P2	98.81 (4)
P1—Ag—P2	122.391 (19)

**Fig. 1.**
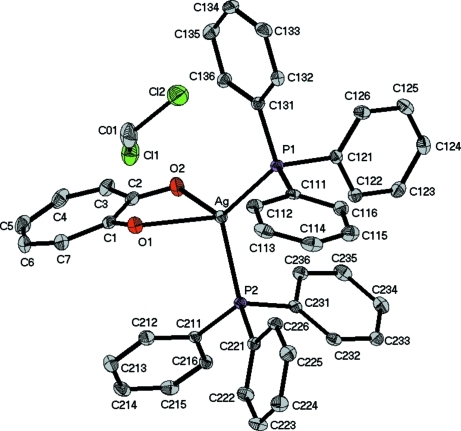
Molecular structure of the title compound. Displacement ellipsoids are drawn at the 50% probability level. H atoms have been omitted for clarity.

## supplementary crystallographic information

### Comment

Tropolone type compounds have been of interest since its first discovery in the early 1940's (Dewar, 1945), with applications in pharmacology (Hill & Steyl, 2008) and catalysis (Crous *et al.*, 2005). Although this type of compounds have been extensively studied, to date no structural data exist, which contain both a tropolone moiety and a silver metal centre. In this regard, we present a bis(triphenylphosphine)silver(I) complex of tropolone.

The Ag—O and Ag—P bond distances differ significantly from each other (Table 1). The distortion of these bonds are also reflected in the interplanar angle between the two planes defined by P—Ag—P and O—Ag—O in the order of 84.90 (4)°. A similar oxalate structure with two Ag centres has been reported (Dean *et al.*, 2001). In this complex the P—Ag—P and O—Ag—O bond angles are 126.35 and 71.28 °, respectively. These values compared to the title compound are significantly larger, indicating the structural effect of the tropolonate moiety on the complexes. In previous studies it has been shown that the tropolonate moiety can act as either a bidentate or a monodentate ligand in its coordination behaviour (Steyl, 2006). Effectively, the tropolonate moiety assumes an optimal coordination mode to pack as efficiently as possible. No classical hydrogen bonds are observed in the solid-state structure of the title compound, although weak interactions do exist between the dichloromethane solvate and the silver complex.

### Experimental

The title complex was synthesized by the addition of sodium salt of tropolone (0.083 g, 0.57 mmol) to a dichloromethane solution (10 ml) of [Cu(PPh_3_)_2_]NO_3_ (0.374 g, 0.287 mmol). On slow evaporation of the solvent, crystals suitable for X-ray crystallography were obtained (yield 85%).

### Refinement

H atoms were positioned geometrically and refined using a riding model, with C—H = 0.95 (CH) and 0.99 (CH_2_) Å and with *U*_iso_(H) = 1.2*U*_eq_(C).

### Crystal data

**Table:**

[Ag(C_7_H_5_O_2_)(C_18_H_15_P)_2_]·CH_2_Cl_2_	*Z* = 2
*M**_r_* = 838.45	*F*(000) = 856
Triclinic, *P*1	*D*_x_ = 1.490 Mg m^−^^3^
Hall symbol: -P 1	Mo *K*α radiation, λ = 0.71073 Å
*a* = 12.0175 (3) Å	Cell parameters from 9106 reflections
*b* = 12.9925 (4) Å	θ = 2.3–28.3°
*c* = 13.8394 (7) Å	µ = 0.81 mm^−^^1^
α = 100.487 (2)°	*T* = 100 K
β = 93.760 (2)°	Cuboid, yellow
γ = 116.809 (1)°	0.19 × 0.11 × 0.08 mm
*V* = 1869.33 (12) Å^3^	

### Data collection

**Table:**

Bruker APEXII CCD diffractometer	8178 independent reflections
Radiation source: fine-focus sealed tube	6931 reflections with *I* > 2σ(*I*)
graphite	*R*_int_ = 0.046
φ and ω scans	θ_max_ = 27.0°, θ_min_ = 1.8°
Absorption correction: multi-scan (*SADABS*; Bruker, 2001)	*h* = −13→15
*T*_min_ = 0.862, *T*_max_ = 0.938	*k* = −16→16
33544 measured reflections	*l* = −17→17

### Refinement

**Table:**

Refinement on *F*^2^	Primary atom site location: structure-invariant direct methods
Least-squares matrix: full	Secondary atom site location: difference Fourier map
*R*[*F*^2^ > 2σ(*F*^2^)] = 0.030	Hydrogen site location: inferred from neighbouring sites
*wR*(*F*^2^) = 0.070	H-atom parameters constrained
*S* = 1.05	*w* = 1/[σ^2^(*F*_o_^2^) + (0.0283*P*)^2^ + 0.6452*P*] where *P* = (*F*_o_^2^ + 2*F*_c_^2^)/3
8178 reflections	(Δ/σ)_max_ = 0.002
460 parameters	Δρ_max_ = 0.51 e Å^−^^3^
0 restraints	Δρ_min_ = −0.41 e Å^−^^3^

### Fractional atomic coordinates and isotropic or equivalent isotropic displacement parameters (Å^2^)

**Table:**

	*x*	*y*	*z*	*U*_iso_*/*U*_eq_	
Ag	0.743570 (16)	0.720243 (14)	0.744274 (12)	0.01303 (5)	
P2	0.86325 (5)	0.62927 (5)	0.65456 (4)	0.01241 (12)	
P1	0.71316 (5)	0.87540 (5)	0.69503 (4)	0.01207 (12)	
O1	0.79674 (15)	0.70564 (14)	0.90673 (11)	0.0184 (4)	
C236	0.6628 (2)	0.49295 (19)	0.49875 (17)	0.0156 (5)	
H236	0.6130	0.4770	0.5501	0.019*	
O2	0.57859 (15)	0.56103 (15)	0.78664 (12)	0.0223 (4)	
C115	1.0246 (2)	1.1352 (2)	0.61838 (19)	0.0231 (6)	
H115	1.0505	1.1622	0.5603	0.028*	
C211	0.8758 (2)	0.50995 (19)	0.69797 (16)	0.0132 (5)	
C131	0.6271 (2)	0.93724 (19)	0.76747 (16)	0.0134 (5)	
C116	0.9035 (2)	1.0442 (2)	0.61294 (17)	0.0174 (5)	
H116	0.8465	1.0099	0.5514	0.021*	
C111	0.8651 (2)	1.00316 (19)	0.69726 (16)	0.0131 (5)	
C132	0.6443 (2)	1.0510 (2)	0.77170 (16)	0.0160 (5)	
H132	0.7057	1.1010	0.7383	0.019*	
C231	0.7939 (2)	0.56327 (18)	0.52340 (16)	0.0119 (4)	
C233	0.8062 (2)	0.5380 (2)	0.34768 (17)	0.0164 (5)	
H233	0.8555	0.5529	0.2960	0.020*	
C232	0.8649 (2)	0.58525 (19)	0.44675 (16)	0.0145 (5)	
H232	0.9542	0.6329	0.4624	0.017*	
C122	0.6516 (2)	0.7563 (2)	0.49517 (16)	0.0165 (5)	
H122	0.7103	0.7299	0.5131	0.020*	
C2	0.6043 (2)	0.5279 (2)	0.86135 (16)	0.0163 (5)	
C121	0.6314 (2)	0.83422 (19)	0.56701 (16)	0.0130 (4)	
C216	0.8462 (2)	0.40129 (19)	0.63496 (17)	0.0149 (5)	
H216	0.8168	0.3862	0.5659	0.018*	
C221	1.0266 (2)	0.73307 (19)	0.65092 (15)	0.0124 (4)	
C133	0.5721 (2)	1.0925 (2)	0.82465 (17)	0.0211 (5)	
H133	0.5848	1.1708	0.8280	0.025*	
C235	0.6052 (2)	0.4466 (2)	0.40016 (18)	0.0187 (5)	
H235	0.5160	0.3985	0.3842	0.022*	
C123	0.5874 (2)	0.7175 (2)	0.39882 (17)	0.0188 (5)	
H123	0.6026	0.6652	0.3507	0.023*	
C226	1.0571 (2)	0.8503 (2)	0.65170 (16)	0.0155 (5)	
H226	0.9939	0.8749	0.6565	0.019*	
C124	0.5006 (2)	0.7547 (2)	0.37165 (17)	0.0200 (5)	
H124	0.4555	0.7270	0.3054	0.024*	
C136	0.5374 (2)	0.8647 (2)	0.81740 (16)	0.0170 (5)	
H136	0.5262	0.7873	0.8164	0.020*	
C222	1.1212 (2)	0.6993 (2)	0.64488 (17)	0.0182 (5)	
H222	1.1019	0.6200	0.6452	0.022*	
C215	0.8596 (2)	0.3150 (2)	0.67277 (18)	0.0191 (5)	
H215	0.8393	0.2410	0.6294	0.023*	
C126	0.5451 (2)	0.8718 (2)	0.53879 (17)	0.0169 (5)	
H126	0.5305	0.9250	0.5864	0.020*	
C114	1.1078 (2)	1.1868 (2)	0.7076 (2)	0.0233 (6)	
H114	1.1902	1.2499	0.7111	0.028*	
C224	1.2722 (2)	0.8965 (2)	0.63878 (18)	0.0203 (5)	
H224	1.3557	0.9522	0.6344	0.024*	
C112	0.9504 (2)	1.0542 (2)	0.78683 (17)	0.0183 (5)	
H112	0.9258	1.0258	0.8446	0.022*	
C113	1.0710 (2)	1.1464 (2)	0.79192 (19)	0.0227 (5)	
H113	1.1282	1.1818	0.8533	0.027*	
C223	1.2432 (2)	0.7803 (2)	0.63844 (18)	0.0203 (5)	
H223	1.3068	0.7561	0.6338	0.024*	
C1	0.7289 (2)	0.6063 (2)	0.92667 (16)	0.0152 (5)	
C134	0.4822 (2)	1.0193 (2)	0.87219 (17)	0.0227 (6)	
H134	0.4322	1.0470	0.9077	0.027*	
C125	0.4804 (2)	0.8322 (2)	0.44196 (18)	0.0198 (5)	
H125	0.4218	0.8585	0.4236	0.024*	
C234	0.6762 (2)	0.4693 (2)	0.32393 (17)	0.0178 (5)	
H234	0.6358	0.4381	0.2562	0.021*	
C3	0.5113 (2)	0.4195 (2)	0.87918 (17)	0.0200 (5)	
H3	0.4331	0.3847	0.8350	0.024*	
C7	0.7775 (2)	0.5754 (2)	1.00616 (17)	0.0207 (5)	
H7	0.8607	0.6334	1.0379	0.025*	
C213	0.9307 (2)	0.4435 (2)	0.83629 (18)	0.0224 (5)	
H213	0.9596	0.4579	0.9054	0.027*	
C225	1.1789 (2)	0.9311 (2)	0.64553 (17)	0.0191 (5)	
H225	1.1987	1.0107	0.6459	0.023*	
C135	0.4643 (2)	0.9060 (2)	0.86865 (18)	0.0239 (6)	
H135	0.4018	0.8560	0.9014	0.029*	
C214	0.9023 (2)	0.3358 (2)	0.77313 (18)	0.0208 (5)	
H214	0.9122	0.2767	0.7987	0.025*	
C212	0.9173 (2)	0.5296 (2)	0.79969 (17)	0.0192 (5)	
H212	0.9363	0.6028	0.8437	0.023*	
C6	0.7276 (3)	0.4775 (2)	1.04662 (18)	0.0258 (6)	
H6	0.7831	0.4780	1.0991	0.031*	
C4	0.5130 (3)	0.3559 (2)	0.94783 (18)	0.0244 (6)	
H4	0.4364	0.2854	0.9435	0.029*	
C5	0.6098 (3)	0.3790 (2)	1.02256 (19)	0.0281 (6)	
H5	0.5933	0.3215	1.0608	0.034*	
Cl1	0.82465 (6)	0.96237 (6)	0.01672 (5)	0.03396 (16)	
C01	0.9005 (3)	1.1161 (3)	0.0739 (2)	0.0347 (7)	
H01A	0.8877	1.1276	0.1442	0.042*	
H01B	0.9926	1.1495	0.0736	0.042*	
Cl2	0.84113 (7)	1.19298 (7)	0.01169 (5)	0.03632 (17)	

### Atomic displacement parameters (Å^2^)

**Table:**

	*U*^11^	*U*^22^	*U*^33^	*U*^12^	*U*^13^	*U*^23^
Ag	0.01416 (9)	0.01288 (9)	0.01448 (9)	0.00751 (7)	0.00373 (7)	0.00533 (6)
P2	0.0128 (3)	0.0121 (3)	0.0143 (3)	0.0071 (2)	0.0035 (2)	0.0041 (2)
P1	0.0126 (3)	0.0120 (3)	0.0140 (3)	0.0069 (2)	0.0042 (2)	0.0046 (2)
O1	0.0174 (9)	0.0154 (8)	0.0183 (8)	0.0046 (7)	0.0003 (7)	0.0043 (7)
C236	0.0139 (12)	0.0145 (12)	0.0195 (12)	0.0066 (10)	0.0044 (9)	0.0067 (9)
O2	0.0160 (9)	0.0265 (10)	0.0211 (9)	0.0052 (8)	0.0008 (7)	0.0123 (7)
C115	0.0213 (14)	0.0205 (13)	0.0322 (14)	0.0099 (11)	0.0124 (11)	0.0135 (11)
C211	0.0109 (11)	0.0157 (12)	0.0168 (11)	0.0077 (10)	0.0054 (9)	0.0081 (9)
C131	0.0132 (11)	0.0142 (11)	0.0121 (11)	0.0068 (10)	0.0015 (9)	0.0014 (9)
C116	0.0196 (13)	0.0154 (12)	0.0200 (12)	0.0096 (10)	0.0049 (10)	0.0070 (10)
C111	0.0123 (11)	0.0113 (11)	0.0192 (11)	0.0083 (9)	0.0043 (9)	0.0039 (9)
C132	0.0171 (12)	0.0176 (12)	0.0141 (11)	0.0085 (10)	0.0021 (9)	0.0045 (9)
C231	0.0153 (11)	0.0078 (10)	0.0147 (11)	0.0072 (9)	0.0017 (9)	0.0027 (8)
C233	0.0185 (12)	0.0166 (12)	0.0179 (11)	0.0104 (10)	0.0049 (9)	0.0063 (9)
C232	0.0120 (11)	0.0120 (11)	0.0210 (12)	0.0062 (10)	0.0030 (9)	0.0056 (9)
C122	0.0170 (12)	0.0182 (12)	0.0180 (12)	0.0099 (10)	0.0054 (9)	0.0077 (9)
C2	0.0186 (12)	0.0181 (12)	0.0149 (11)	0.0102 (10)	0.0066 (9)	0.0049 (9)
C121	0.0111 (11)	0.0124 (11)	0.0145 (11)	0.0038 (9)	0.0025 (9)	0.0057 (9)
C216	0.0141 (12)	0.0159 (12)	0.0167 (11)	0.0073 (10)	0.0039 (9)	0.0071 (9)
C221	0.0128 (11)	0.0117 (11)	0.0117 (10)	0.0056 (9)	0.0012 (9)	0.0012 (8)
C133	0.0264 (14)	0.0221 (13)	0.0207 (12)	0.0179 (12)	0.0012 (10)	0.0018 (10)
C235	0.0118 (12)	0.0137 (12)	0.0262 (13)	0.0028 (10)	−0.0008 (10)	0.0048 (10)
C123	0.0219 (13)	0.0179 (12)	0.0177 (12)	0.0093 (11)	0.0072 (10)	0.0056 (10)
C226	0.0162 (12)	0.0159 (12)	0.0184 (11)	0.0105 (10)	0.0041 (9)	0.0049 (9)
C124	0.0206 (13)	0.0200 (13)	0.0155 (12)	0.0063 (11)	−0.0003 (10)	0.0054 (10)
C136	0.0189 (12)	0.0171 (12)	0.0175 (11)	0.0091 (10)	0.0067 (10)	0.0068 (9)
C222	0.0179 (12)	0.0139 (12)	0.0236 (12)	0.0088 (10)	0.0027 (10)	0.0034 (10)
C215	0.0166 (12)	0.0131 (12)	0.0274 (13)	0.0068 (10)	0.0053 (10)	0.0042 (10)
C126	0.0161 (12)	0.0144 (12)	0.0211 (12)	0.0081 (10)	0.0037 (10)	0.0036 (9)
C114	0.0138 (13)	0.0137 (12)	0.0420 (16)	0.0049 (10)	0.0077 (11)	0.0092 (11)
C224	0.0138 (12)	0.0197 (13)	0.0233 (13)	0.0045 (10)	0.0028 (10)	0.0051 (10)
C112	0.0179 (12)	0.0173 (12)	0.0216 (12)	0.0095 (11)	0.0053 (10)	0.0050 (10)
C113	0.0193 (13)	0.0171 (13)	0.0306 (14)	0.0102 (11)	−0.0020 (11)	0.0008 (10)
C223	0.0152 (12)	0.0211 (13)	0.0278 (13)	0.0113 (11)	0.0031 (10)	0.0056 (10)
C1	0.0202 (12)	0.0164 (12)	0.0128 (11)	0.0118 (10)	0.0055 (9)	0.0026 (9)
C134	0.0240 (14)	0.0336 (15)	0.0175 (12)	0.0202 (12)	0.0061 (10)	0.0033 (11)
C125	0.0161 (12)	0.0200 (13)	0.0241 (13)	0.0092 (11)	−0.0009 (10)	0.0065 (10)
C234	0.0209 (13)	0.0132 (12)	0.0178 (12)	0.0085 (10)	−0.0028 (10)	0.0008 (9)
C3	0.0211 (13)	0.0193 (13)	0.0161 (12)	0.0071 (11)	0.0045 (10)	0.0025 (10)
C7	0.0210 (13)	0.0271 (14)	0.0165 (12)	0.0131 (11)	0.0029 (10)	0.0063 (10)
C213	0.0232 (14)	0.0280 (14)	0.0205 (12)	0.0142 (12)	0.0031 (10)	0.0100 (11)
C225	0.0223 (13)	0.0136 (12)	0.0224 (12)	0.0087 (11)	0.0055 (10)	0.0052 (10)
C135	0.0216 (14)	0.0314 (15)	0.0218 (13)	0.0133 (12)	0.0095 (11)	0.0092 (11)
C214	0.0173 (13)	0.0207 (13)	0.0306 (14)	0.0107 (11)	0.0069 (11)	0.0146 (11)
C212	0.0222 (13)	0.0202 (13)	0.0182 (12)	0.0130 (11)	0.0039 (10)	0.0034 (10)
C6	0.0353 (16)	0.0388 (16)	0.0164 (12)	0.0257 (14)	0.0078 (11)	0.0134 (11)
C4	0.0320 (15)	0.0166 (13)	0.0232 (13)	0.0085 (12)	0.0139 (12)	0.0065 (10)
C5	0.0455 (18)	0.0265 (15)	0.0265 (14)	0.0234 (14)	0.0185 (13)	0.0173 (12)
Cl1	0.0284 (4)	0.0349 (4)	0.0261 (3)	0.0032 (3)	0.0014 (3)	0.0117 (3)
C01	0.0299 (16)	0.0410 (17)	0.0203 (14)	0.0081 (14)	−0.0073 (11)	0.0050 (12)
Cl2	0.0302 (4)	0.0453 (4)	0.0321 (4)	0.0184 (3)	0.0015 (3)	0.0057 (3)

### Geometric parameters (Å, °)

**Table:**

Ag—O1	2.3612 (16)	C123—H123	0.9500
Ag—O2	2.3342 (16)	C226—C225	1.384 (3)
Ag—P1	2.4070 (6)	C226—H226	0.9500
Ag—P2	2.4981 (5)	C124—C125	1.384 (3)
P2—C231	1.821 (2)	C124—H124	0.9500
P2—C221	1.823 (2)	C136—C135	1.389 (3)
P2—C211	1.824 (2)	C136—H136	0.9500
P1—C131	1.820 (2)	C222—C223	1.387 (3)
P1—C111	1.825 (2)	C222—H222	0.9500
P1—C121	1.829 (2)	C215—C214	1.382 (3)
O1—C1	1.271 (3)	C215—H215	0.9500
C236—C235	1.380 (3)	C126—C125	1.385 (3)
C236—C231	1.397 (3)	C126—H126	0.9500
C236—H236	0.9500	C114—C113	1.383 (3)
O2—C2	1.261 (3)	C114—H114	0.9500
C115—C114	1.381 (4)	C224—C225	1.385 (3)
C115—C116	1.386 (3)	C224—C223	1.388 (3)
C115—H115	0.9500	C224—H224	0.9500
C211—C216	1.392 (3)	C112—C113	1.390 (3)
C211—C212	1.399 (3)	C112—H112	0.9500
C131—C132	1.386 (3)	C113—H113	0.9500
C131—C136	1.395 (3)	C223—H223	0.9500
C116—C111	1.391 (3)	C1—C7	1.414 (3)
C116—H116	0.9500	C134—C135	1.379 (3)
C111—C112	1.395 (3)	C134—H134	0.9500
C132—C133	1.393 (3)	C125—H125	0.9500
C132—H132	0.9500	C234—H234	0.9500
C231—C232	1.395 (3)	C3—C4	1.373 (3)
C233—C234	1.383 (3)	C3—H3	0.9500
C233—C232	1.390 (3)	C7—C6	1.382 (3)
C233—H233	0.9500	C7—H7	0.9500
C232—H232	0.9500	C213—C212	1.376 (3)
C122—C123	1.377 (3)	C213—C214	1.387 (3)
C122—C121	1.400 (3)	C213—H213	0.9500
C122—H122	0.9500	C225—H225	0.9500
C2—C3	1.427 (3)	C135—H135	0.9500
C2—C1	1.486 (3)	C214—H214	0.9500
C121—C126	1.391 (3)	C212—H212	0.9500
C216—C215	1.387 (3)	C6—C5	1.379 (4)
C216—H216	0.9500	C6—H6	0.9500
C221—C222	1.393 (3)	C4—C5	1.388 (4)
C221—C226	1.394 (3)	C4—H4	0.9500
C133—C134	1.378 (3)	C5—H5	0.9500
C133—H133	0.9500	Cl1—C01	1.770 (3)
C235—C234	1.390 (3)	C01—Cl2	1.768 (3)
C235—H235	0.9500	C01—H01A	0.9900
C123—C124	1.390 (3)	C01—H01B	0.9900
			
O2—Ag—O1	68.67 (6)	C125—C124—H124	120.3
O2—Ag—P1	121.43 (4)	C123—C124—H124	120.3
O1—Ag—P1	128.35 (4)	C135—C136—C131	119.8 (2)
O2—Ag—P2	103.97 (4)	C135—C136—H136	120.1
O1—Ag—P2	98.81 (4)	C131—C136—H136	120.1
P1—Ag—P2	122.391 (19)	C223—C222—C221	120.7 (2)
C231—P2—C221	103.35 (10)	C223—C222—H222	119.6
C231—P2—C211	103.57 (10)	C221—C222—H222	119.6
C221—P2—C211	103.62 (10)	C214—C215—C216	120.5 (2)
C231—P2—Ag	111.93 (7)	C214—C215—H215	119.8
C221—P2—Ag	114.95 (7)	C216—C215—H215	119.8
C211—P2—Ag	117.80 (7)	C125—C126—C121	120.6 (2)
C131—P1—C111	104.60 (10)	C125—C126—H126	119.7
C131—P1—C121	103.57 (10)	C121—C126—H126	119.7
C111—P1—C121	104.03 (10)	C115—C114—C113	119.9 (2)
C131—P1—Ag	118.60 (7)	C115—C114—H114	120.1
C111—P1—Ag	110.63 (7)	C113—C114—H114	120.1
C121—P1—Ag	114.02 (7)	C225—C224—C223	119.7 (2)
C1—O1—Ag	115.51 (14)	C225—C224—H224	120.1
C235—C236—C231	120.3 (2)	C223—C224—H224	120.1
C235—C236—H236	119.8	C113—C112—C111	120.3 (2)
C231—C236—H236	119.8	C113—C112—H112	119.8
C2—O2—Ag	117.18 (14)	C111—C112—H112	119.8
C114—C115—C116	120.4 (2)	C114—C113—C112	120.0 (2)
C114—C115—H115	119.8	C114—C113—H113	120.0
C116—C115—H115	119.8	C112—C113—H113	120.0
C216—C211—C212	119.0 (2)	C222—C223—C224	119.9 (2)
C216—C211—P2	123.18 (17)	C222—C223—H223	120.0
C212—C211—P2	117.83 (17)	C224—C223—H223	120.0
C132—C131—C136	119.41 (19)	O1—C1—C7	118.7 (2)
C132—C131—P1	122.58 (16)	O1—C1—C2	117.0 (2)
C136—C131—P1	117.97 (16)	C7—C1—C2	124.2 (2)
C115—C116—C111	120.2 (2)	C133—C134—C135	120.5 (2)
C115—C116—H116	119.9	C133—C134—H134	119.7
C111—C116—H116	119.9	C135—C134—H134	119.7
C116—C111—C112	119.1 (2)	C124—C125—C126	120.4 (2)
C116—C111—P1	123.35 (18)	C124—C125—H125	119.8
C112—C111—P1	117.30 (17)	C126—C125—H125	119.8
C131—C132—C133	120.4 (2)	C233—C234—C235	119.3 (2)
C131—C132—H132	119.8	C233—C234—H234	120.3
C133—C132—H132	119.8	C235—C234—H234	120.3
C232—C231—C236	118.8 (2)	C4—C3—C2	132.4 (2)
C232—C231—P2	122.87 (17)	C4—C3—H3	113.8
C236—C231—P2	118.21 (16)	C2—C3—H3	113.8
C234—C233—C232	120.3 (2)	C6—C7—C1	132.6 (2)
C234—C233—H233	119.8	C6—C7—H7	113.7
C232—C233—H233	119.8	C1—C7—H7	113.7
C233—C232—C231	120.5 (2)	C212—C213—C214	120.5 (2)
C233—C232—H232	119.8	C212—C213—H213	119.7
C231—C232—H232	119.8	C214—C213—H213	119.7
C123—C122—C121	120.7 (2)	C226—C225—C224	120.4 (2)
C123—C122—H122	119.6	C226—C225—H225	119.8
C121—C122—H122	119.6	C224—C225—H225	119.8
O2—C2—C3	118.2 (2)	C134—C135—C136	120.2 (2)
O2—C2—C1	117.6 (2)	C134—C135—H135	119.9
C3—C2—C1	124.2 (2)	C136—C135—H135	119.9
C126—C121—C122	118.5 (2)	C215—C214—C213	119.5 (2)
C126—C121—P1	123.10 (16)	C215—C214—H214	120.2
C122—C121—P1	118.31 (17)	C213—C214—H214	120.2
C215—C216—C211	120.2 (2)	C213—C212—C211	120.3 (2)
C215—C216—H216	119.9	C213—C212—H212	119.8
C211—C216—H216	119.9	C211—C212—H212	119.8
C222—C221—C226	118.8 (2)	C5—C6—C7	130.0 (3)
C222—C221—P2	122.69 (17)	C5—C6—H6	115.0
C226—C221—P2	118.54 (16)	C7—C6—H6	115.0
C134—C133—C132	119.6 (2)	C3—C4—C5	129.8 (2)
C134—C133—H133	120.2	C3—C4—H4	115.1
C132—C133—H133	120.2	C5—C4—H4	115.1
C236—C235—C234	120.8 (2)	C6—C5—C4	126.3 (2)
C236—C235—H235	119.6	C6—C5—H5	116.9
C234—C235—H235	119.6	C4—C5—H5	116.9
C122—C123—C124	120.3 (2)	Cl2—C01—Cl1	111.60 (15)
C122—C123—H123	119.8	Cl2—C01—H01A	109.3
C124—C123—H123	119.8	Cl1—C01—H01A	109.3
C225—C226—C221	120.5 (2)	Cl2—C01—H01B	109.3
C225—C226—H226	119.7	Cl1—C01—H01B	109.3
C221—C226—H226	119.7	H01A—C01—H01B	108.0
C125—C124—C123	119.4 (2)		
			
O2—Ag—P2—C231	83.92 (9)	C131—P1—C121—C126	13.0 (2)
O1—Ag—P2—C231	153.99 (8)	C111—P1—C121—C126	−96.14 (19)
P1—Ag—P2—C231	−58.66 (8)	Ag—P1—C121—C126	143.27 (17)
O2—Ag—P2—C221	−158.57 (9)	C131—P1—C121—C122	−163.60 (17)
O1—Ag—P2—C221	−88.49 (9)	C111—P1—C121—C122	87.28 (18)
P1—Ag—P2—C221	58.85 (8)	Ag—P1—C121—C122	−33.31 (19)
O2—Ag—P2—C211	−35.96 (10)	C212—C211—C216—C215	−0.9 (3)
O1—Ag—P2—C211	34.11 (10)	P2—C211—C216—C215	179.11 (16)
P1—Ag—P2—C211	−178.54 (9)	C231—P2—C221—C222	−86.0 (2)
O2—Ag—P1—C131	44.96 (10)	C211—P2—C221—C222	21.8 (2)
O1—Ag—P1—C131	−41.59 (10)	Ag—P2—C221—C222	151.70 (16)
P2—Ag—P1—C131	−178.76 (8)	C231—P2—C221—C226	92.32 (18)
O2—Ag—P1—C111	165.75 (9)	C211—P2—C221—C226	−159.88 (17)
O1—Ag—P1—C111	79.20 (9)	Ag—P2—C221—C226	−29.94 (19)
P2—Ag—P1—C111	−57.97 (8)	C131—C132—C133—C134	−0.7 (4)
O2—Ag—P1—C121	−77.42 (9)	C231—C236—C235—C234	−0.3 (3)
O1—Ag—P1—C121	−163.97 (8)	C121—C122—C123—C124	−0.5 (3)
P2—Ag—P1—C121	58.86 (8)	C222—C221—C226—C225	0.7 (3)
O2—Ag—O1—C1	17.65 (14)	P2—C221—C226—C225	−177.75 (17)
P1—Ag—O1—C1	131.53 (13)	C122—C123—C124—C125	0.9 (3)
P2—Ag—O1—C1	−83.99 (14)	C132—C131—C136—C135	1.5 (3)
O1—Ag—O2—C2	−15.03 (15)	P1—C131—C136—C135	−176.21 (19)
P1—Ag—O2—C2	−137.85 (14)	C226—C221—C222—C223	−0.8 (3)
P2—Ag—O2—C2	79.12 (15)	P2—C221—C222—C223	177.51 (18)
C231—P2—C211—C216	5.0 (2)	C211—C216—C215—C214	0.0 (3)
C221—P2—C211—C216	−102.68 (19)	C122—C121—C126—C125	0.4 (3)
Ag—P2—C211—C216	129.12 (16)	P1—C121—C126—C125	−176.20 (18)
C231—P2—C211—C212	−175.02 (17)	C116—C115—C114—C113	1.0 (4)
C221—P2—C211—C212	77.34 (18)	C116—C111—C112—C113	1.3 (3)
Ag—P2—C211—C212	−50.86 (19)	P1—C111—C112—C113	175.55 (17)
C111—P1—C131—C132	30.9 (2)	C115—C114—C113—C112	−0.1 (3)
C121—P1—C131—C132	−77.8 (2)	C111—C112—C113—C114	−1.1 (3)
Ag—P1—C131—C132	154.70 (17)	C221—C222—C223—C224	0.5 (4)
C111—P1—C131—C136	−151.45 (18)	C225—C224—C223—C222	0.0 (4)
C121—P1—C131—C136	99.85 (19)	Ag—O1—C1—C7	158.97 (15)
Ag—P1—C131—C136	−27.6 (2)	Ag—O1—C1—C2	−18.7 (2)
C114—C115—C116—C111	−0.7 (3)	O2—C2—C1—O1	5.2 (3)
C115—C116—C111—C112	−0.4 (3)	C3—C2—C1—O1	−173.26 (19)
C115—C116—C111—P1	−174.29 (17)	O2—C2—C1—C7	−172.4 (2)
C131—P1—C111—C116	−113.66 (19)	C3—C2—C1—C7	9.2 (3)
C121—P1—C111—C116	−5.3 (2)	C132—C133—C134—C135	0.8 (4)
Ag—P1—C111—C116	117.54 (17)	C123—C124—C125—C126	−0.6 (4)
C131—P1—C111—C112	72.36 (18)	C121—C126—C125—C124	0.0 (4)
C121—P1—C111—C112	−179.28 (16)	C232—C233—C234—C235	−1.0 (3)
Ag—P1—C111—C112	−56.44 (17)	C236—C235—C234—C233	0.9 (3)
C136—C131—C132—C133	−0.5 (3)	O2—C2—C3—C4	174.6 (2)
P1—C131—C132—C133	177.16 (18)	C1—C2—C3—C4	−7.0 (4)
C235—C236—C231—C232	−0.2 (3)	O1—C1—C7—C6	178.7 (2)
C235—C236—C231—P2	176.33 (17)	C2—C1—C7—C6	−3.8 (4)
C221—P2—C231—C232	6.6 (2)	C221—C226—C225—C224	−0.2 (3)
C211—P2—C231—C232	−101.18 (19)	C223—C224—C225—C226	−0.1 (4)
Ag—P2—C231—C232	130.91 (16)	C133—C134—C135—C136	0.3 (4)
C221—P2—C231—C236	−169.69 (17)	C131—C136—C135—C134	−1.5 (4)
C211—P2—C231—C236	82.48 (18)	C216—C215—C214—C213	0.6 (3)
Ag—P2—C231—C236	−45.43 (19)	C212—C213—C214—C215	−0.4 (3)
C234—C233—C232—C231	0.6 (3)	C214—C213—C212—C211	−0.5 (3)
C236—C231—C232—C233	0.0 (3)	C216—C211—C212—C213	1.2 (3)
P2—C231—C232—C233	−176.29 (17)	P2—C211—C212—C213	−178.86 (17)
Ag—O2—C2—C3	−169.93 (14)	C1—C7—C6—C5	−2.5 (4)
Ag—O2—C2—C1	11.5 (2)	C2—C3—C4—C5	−0.4 (4)
C123—C122—C121—C126	−0.1 (3)	C7—C6—C5—C4	1.3 (4)
C123—C122—C121—P1	176.62 (17)	C3—C4—C5—C6	2.8 (4)
